# Reduction of the survival time of pig xenotransplants by porcine cytomegalovirus

**DOI:** 10.1186/s12985-018-1088-2

**Published:** 2018-11-08

**Authors:** Joachim Denner

**Affiliations:** Robert Koch Fellow, Robert Koch Institute, Nordufer 20, 13353 Berlin, Germany

**Keywords:** Porcine cytomegalovirus, Xenotransplantation, Herpesviruses

## Abstract

**Background:**

Xenotransplantation using pig cells, tissues and organs may help to overcome the shortage of human tissues and organs for the treatment of tissue and organ failure. Progress in the prevention of immunological rejection using genetically modified pigs and new, more effective, immunosuppression regimens will allow clinical application of xenotransplantation in near future. However, xenotransplantation may be associated with the transmission of potentially zoonotic porcine microorganisms. Until now the only xenotransplantation-associated transmission was the transmission of the porcine cytomegalovirus (PCMV) into non-human primates. PCMV caused a significant reduction of the survival time of the pig transplant.

**Main body of the abstract:**

Here the available publications were analysed in order to establish the mechanism how PCMV shortened the survival time of xenotransplants. PCMV is a herpesvirus related to the human cytomegalovirus and the human herpesviruses 6 and 7. These three human herpesviruses can cause serious disease among immunocompromised human individuals, including transplant recipients. It was shown that PCMV predominantly contributes to the reduction of transplant survival in non-human primates by disruption of the coagulation system and by suppression and exhaustion of the immune system.

**Conclusion:**

Although it is still unknown whether PCMV infects primate cells including human cells, indirect mechanism of the virus infection may cause reduction of the xenotransplant survival in future clinical trials and therefore PCMV has to be eliminated from donor pigs.

## Background

In the last years, a significant progress was achieved in the development of xenotransplantation [1–4]. The main achievements were the generation of pigs with multiple genetic modifications [5–7] and new, more efficient immunosuppression regimens [8, 9], allowing to overcome the hyperacute rejection as well as other immunological reactions. Increased survival times of different pig transplants in non-human primates demonstrated the impressive progress made in the field (Table 1) [3, 8–17]. However, immunological rejection and physiological incompatibility are only two hurdles on the way to the clinical application of xenotransplantation. Another hurdle is the risk to transmit porcine microorganisms to the recipient which may induce severe disease (zoonosis or xenosis). Microorganisms include bacteria, protozoa, fungi and viruses. In this context viruses are certainly the most harmful microorganisms due to the lack of effective antivirals and vaccines. Among the porcine viruses of interest are DNA viruses such as PCMV, the porcine circoviruses 1, 2 and 3 (PCV1, PCV2, PCV3), and the porcine lymphotropic herpesviruses (PLHV-1, PLHV-2, PLHV-3) as well as RNA viruses such as the hepatitis E virus (HEV), the porcine reproductive and respiratory syndrome virus (PRRSV) and the Nipah virus (NIV) (for reviews discussing each virus type in the context of xenotransplantation see [18–25]). Some of these viruses, e.g. PCV2, PRRSV and NIV, cause severe disease in the infected pigs and can easily be detected. For the detection of the viruses not causing diseases in the infected pigs sensitive screening methods are required. Among the viruses with potential zoonotic potential are also the porcine endogenous retroviruses (PERVs) which are integrated in the genome of all pigs and which - in contrast to all other porcine viruses, bacteria and fungi - cannot be eliminated by selection of negative animals, vaccination, treatment, early weaning, colostrum deprivation, Caesarean delivery or embryo transfer [26]. This manuscript will concentrate on the mechanism how PCMV contributes to the reduction of transplant survival.

**Table 1:** Survival time of different porcine xenotransplants in non-human primates

Pig transplant	Longest survival time (days)	Reference
Islet cells	950	Shin et al., [10]
Hearts, heterotopic	945	Mohiuddin et al., [8]
Hearts, orthotopic	57	Byrne et al., [11]
Kidney	499	Higginbotham et al., [9], Iwase et al., [12, 13], Wang et al., [3], Kim et al., [17]
Neurones	549	Badin et al., [14]
Cornea	511	Kim et al., [15]
Liver	29	Wang et al., [3]
Lung	10	Watanabe et al., [16]

## Main text

### Risk evaluation of some porcine viruses

Viruses affecting the health of the donor pigs will harm the transplant and have to be eliminated. For some of these viruses effective vaccines exist, which prevent the infection or at least the outbreak of the disease. More difficult is the situation with viruses not affecting the health of the donor pigs. With two exceptions we do not know whether transmission of these viruses will harm the transplant recipient. On exception is HEV. It was shown that HEV can be transmitted to humans by undercooked meat or contact and that infection of immune-compromised humans leads to a chronic HEV infection, whereas infection of individuals with pre-existing liver disease may be fatal [20, 21]. There are no licensed vaccines or effective antivirals against HEV. The other, more important exception, is PCMV, which will be discussed in the next chapter.

The risk posed by PERV is difficult to evaluate. Retroviruses are known to induce tumours and / or immunodeficiency in the infected individuals. Until now, no transmission of PERV was observed in numerous pig to non-human primate xenotransplantations and in first clinical trials in humans [26, 27]. If recent reports, demonstrating inactivation of all PERVs in pig cells [28] and in live animals [29] by gene editing using clustered regularly interspaced short palindromic repeats/ CRISPR associated (CRISPR/Cas), can be confirmed, the risk posed by PERV will be eliminated [30].

### Special case: PCMV

PCMV is an enveloped DNA virus belonging to the family *Herpesviridae*, subfamily *Betaherpesvirinae*, genus *Roseolovirus* [31]. PCMV is found in the tissues throughout the body including the nose of infected newborn piglets where it causes rhinitis and conjunctivitis. Most infections are sub-clinical, clinical disease is rare [32]. Clinical signs are only seen if PCMV infects a sow for the first time when she is late in pregnancy. Foetal deaths, mummified foetuses, stillbirths, and weak piglets are observed. The virus is shed in discharges from the nose and eyes, urine and farrowing fluids, it is also transmitted via the boar through semen and crosses the placenta to infect piglets before birth [32–34].

Despite the similar name, PCMV is more closely related with human herpesvirus-6 (HHV-6) and HHV-7, which are also Roseoloviruses, but not so closely with the human cytomegalovirus (HCMV), also called HHV-5 [31, 35, 36]. HCMV is the cause of disease in the human fetus, the allograft recipient, AIDS patient, those admitted to intensive care units, and in the elderly [37]. HCMV is the single most important infectious agent affecting recipients of organ transplants, with at least two-thirds of these patients having CMV infection after transplantation [38]. During solid organ transplantation, seropositive donors frequently transmit HCMV to seronegative recipients (primary infection) [38, 39]. Primary infection has the greatest clinical impact. In addition, during allotransplantation HCMV may be re-activated by the allo-immune responses and by the immunosuppression in the HCMV-positive recipient [40]. Finally, superinfections have been described [38]. Pneumonia induced by HCMV is most common after lung and heart/lung transplantations in humans [41, 42], however, the spectrum of CMV pneumonia has changed with the introduction of routine antiviral prophylaxis [43–45]. Consumptive coagulopathy (CC) and thrombotic microangiopathy have been described in disseminated HCMV infection in humans [43, 44]. The thrombotic microangiopathy can be treated by ganciclovir. HCMV produces overt disease only if the viral load increases to high levels. Due to a robust immune response the infected individual usually remains asymptomatic. However, this permanent control of HCMV impairs the immune system and leads to immunosuppression [37]. HCMV carry in its genome the viral gene UL111A, which encodes a viral IL-10 which is homoloque to the cellular IL-10, a well-known immunosuppressive cytokine [45]. This protein is also expressed in healthy HCMV-positive donors and may play a key role in sensing or modifying the host environment during latency [46].

As mentioned, the human roseoloviruses HHV-6A, HHV-6B and HHV-7 are more closely related with PCMV, even cross-reacting antibodies have been observed in humans [47]. HHV-6A, HHV-6B and HHV-7 are widely distributed in the human population. Like other herpesviruses, these viruses cause acute infection, establish latency, and in the case of HHV-6A and HHV-6B, whole virus can integrate into the host chromosome [48]. Primary infection with HHV-6B occurs in nearly all children and was first linked to the clinical syndrome roseola infantum. However, roseolovirus infection results in a spectrum of clinical disease, ranging from asymptomatic infection to acute febrile illnesses with severe neurologic complications. Generally, reactivation of roseoloviruses has been associated with various clinical syndromes including fever, encephalitis, pneumonitis, hepatitis, bone marrow suppression, and a graft versus host disease (GVHD)-like rash, however co-infection with other viruses including HCMV cannot be excluded [49]. HHV-6 and HHV-7 are together with HCMV common infections in transplant recipients and have been clearly associated with early transplant rejection [38, 50–53].

### Reduction of transplant survival time in non-human primates by PCMV

PCMV is the first virus with proven pathogenicity in xenotransplantation. In numerous preclinical trials transplanting pig organs into non-human primates, a significant reduction of the survival time was observed (Table 2) [54–60]. Transplantation of PCMV-positive thymokidneys into baboons resulted in an increased PCMV titre, hematuria, systemic coagulopathy and a reduced survival time of the pig xenotransplant [54].

Transplanting pig kidneys into baboons, the first kidney coming from a PCMV-positive animal failed after 13 days and a second kidney from a PCMV-negative animal was transplanted after excision of the first kidney. The second transplant survived for 60 days without hemorrhagic changes, clearly demonstrating the PCMV infection was the reason of the short survival time of the first transplant [58].

**Table 2:** Xenotransplantations of pig organs with and without PCMV infection

Pig organ (pig race)	Genetic modification	Recipient	PCMV	Survival time without PCMV in days (number of cases)	Survival time with PCMV in days (number of cases)	Comments concerning PCMV infection	BaCMV	References
Thymokidney, (Landrace)	HDAF*	Baboon (*n* = 6)	Up-regulated in all animals except #4	No difference		Intravascular coagulation syndrome in some animals, thrombocytopenia, ureteric necrosis in one animal	In all animals except #5, activation in 3 animals, pneumonitis and death in one animal	Mueller et al., 2002 [54], Gollackner et al., 2003 [55]
Thymokidney, kidney, thymus, heart (Landrace)	HDAF	Baboon (*n* = 22)	In all donors		11–32 (PCMV up-regulation in 15/22 animals) 7–27 (PCMV no upregulation in 7 animals)	Immunosuppressed PCMV positive animal: upregulation of PCMV	Not investigated	Mueller et al., 2004 [56]
Heterotopic heart (Landrace x Large White)	HDAF	Baboons (*n* = 9)	2 positive, 3 negative, 4 negative by early weaning	33 (4 early weaned) 53 (3 negative animals)	20 (2 positive animals)	Consumptive coagulopathy	All positive, no up-regulation due to ganciclovir	Mueller et al., 2004 [57]
Thymokidney, kidney (MGH ^a^ miniature swíne)	GalT-KO**	Baboon (*n* = 21)	8 negative ^a^, 10 positive, 3 negative after Caesarean delivery^b^	48.4 (8 negative)^b^ 53 (3 Caesarean delivery)^c^	14.1 (10 positive)	Enhanced ICAM-1 and MHC class II expression in the transplant (endothelial cell activation)	No BaCMV in one PCMV-negative animal	Yamada et al., 2014 [58]
Kidney	GalT-KO	Cynomolgus monkeys (*n* = 8)	8 negative ^d^, 5 positive ^e^	28.7^d^	9.2^e^		Not applicable	Sekijima et al., 2014 [59]
Liver (MGH ^a^ miniature swine)	GalT-KO	Baboons (*n* = 4)		5, 25, 29	8	Hemorrhagic necrosis, focal PCMV inclusion	Not investigated	Shah et al., 2017 [60]

*HDAF human complement decay-accelerating factor, synonym *CD55* **GalT-KO alpha1,3-galactosyltransferase gene-knockout; ^a^Massachusetts General Hospital; ^b^Naturally *PCMV*-negative; ^c^After Caesarean delivery; ^d^Pigs from the Massachusetts General Hospital/Nippon Institute for Biological Science; ^e^Pigs from the Meji University

Transmission of PCMV was also observed in orthotopic pig heart transplantations in baboons [61, 62]. PCMV-positive cells were found disseminated in the baboon recipient, it seemed likely that these are PCMV-infected pig cells [63]. The influence on the survival time in this setting is still unclear. In this case, replication in the xenotransplant, the pig heart, should be sufficient to generate a high virus load in the blood of the baboon.

### Are herpesviruses species-specific?

At present it is still unclear, whether PCMV can infect human and non-human primate cells. There is one report showing infection of human cells [64] and another not showing infection [65]. Ever since their first isolation, cytomegaloviruses have been recognized as being highly species specific, replicating only in cells of their own or a closely related host species, while cells of phylogenetically more distant hosts are usually not permissive for viral replication. For instance, HCMV replicates in human and chimpanzee fibroblasts but not in rodent cells, and murine cytomegalovirus (MCMV) replicates in cells of mice and rats but not in primate cells [66]. However, MCMV can be adapted stepwise to mutate and replicate in cultured human cells [66]. Because a single gene of the HCMV encoding a mitochondrial inhibitor of apoptosis is sufficient to allow MCMV replication in human cells, induction of apoptosis was thought to serve as an innate immune defence to inhibit cross-species infections of rodent CMVs [67]. MCMV is also thought to be a species-specific virus, however a recombinant MCMV entered and expressed reporter genes in both rat and human brain cells [68]. These data show that under certain conditions cytomegaloviruses may mutate and replicate in other species. In the case of the mutated MCMV able to replicate in human cells only a limited number of mutations was detected [66]. When analysing PCMV in baboons which received a PCMV-infected pig heart, low numbers of PCMV-positive cells were found in all organs of the baboon, suggesting that disseminated pig cells produce the virus found in the blood [63].

Another example for transspecies transmission of a herpesvirus is the baboon cytomegalovirus (BaCMV). This virus was shown to infect human cell in vitro [69]. Furthermore, BaCMV was found in a human recipient after transplantation of a baboon liver and BaCMV able to replicate on human cells was isolated from the patient [70]. Meanwhile it was also shown that HCMV infects pig cells [71].

### Possible mechanisms how PCMV causes reduction of the transplant survival time

Since several reviews concerning the role of PCMV in xenotransplantation have been published [18, 19, 22, 72], here mainly the mechanisms leading to a reduced survival will be put into focus and analysed in more details (see Table 2). There are two main mechanisms: First, the influence on the coagulation system, and, second, the influence on the immune system of the infected animal.

Consumptive coagulopathy (CC) defined by thrombocytopenia, decreasing fibrinogen, and haemorrhage, has been observed in pig to non-human primate xenotransplantations [73–75]. Infections with other herpesviruses have also been shown to directly activate prothrombin and initiate clotting [76, 77]. In vitro activation of the porcine tissue factor (TF) by PCMV infection of porcine aortic endothelial cells (PAEC) was reported, although in vivo no correlation between TF activation and PCMV infection was observed [55]. When pig kidneys were transplanted into baboons, an enhanced intercellular adhesion molecule 1 (ICAM-1) expression was observed in the transplant (endothelial cell activation) [58]. This reminds the situation with HCMV. After in vitro infection of HUVEC (human umbilical vein endothelial cells) with HCMV also an enhanced ICAM-1 expression was observed [78].

PCMV is an immunosuppressive virus modulating the expression of immune-related genes [79]. A PCMV infection in pigs is often associated with opportunistic bacterial infections [35]. Transcriptome analysis of PCMV-infected thymuses showed that numerous immune-regulatory genes were up- or downregulated [80]. When porcine microRNAs (miRNA) were analysed in PCMV-infected and none-infected porcine macrophages, the differentially expressed miRNA were mainly involved in immune and metabolic processes [81].

### PCMV and PERVs

No increased expression of PERV in transplanted pig kidneys from PCMV-positive animals compared with kidneys from uninfected animals was reported [82]. The interaction of PCMV and PERV deserves further investigation, especially in lymphoid tissues. So it was shown that allogenic stimulation [83] or cultivation in culture medium [63] of pig PBMCs significantly enhanced the expression of PCMV. On the other hand, stimulation of pig PBMCs with mitogens induced an enhanced expression of PERV associated with release of virus particles [84–86]. In this context two scenarios are of interest and should be investigated: PERV-expression in PCMV-infected pig immune cells and PERV-expression in pig lymphocytes immune-stimulated by PCMV.

### How to prevent PCMV transmission

Like many of the potentially zoonotic microorganisms, PCMV can be eliminated by selection of PCMV-negative animals or by early weaning. Since PCMV can be transmitted via the placenta, Caesarean section, colostrum deprivation and embryo transfer may be useful. There were some efforts to eliminate PCMV by early weaning [57, 87]. Only recently it was shown that early weaning completely eliminated porcine cytomegalovirus from a newly established facility for pig donors generated for xenotransplantation [88].

Antiviral drugs such as ganciclovir, cidofovir and to a lesser extent the more toxic compounds foscarnet and acyclovir have been shown to inhibit replication of PCMV [89]. However, PCMV – in contrast to the human CMV – is highly resistant to ganciclovir [90]. Unfortunately, vaccines against PCMV are not yet available, although first immunisation experiments with PCMV-derived antigens were successful [91].

## Conclusion

PCMV is the first virus with proven pathogenicity in xenotranplantation. In numerous preclinical trials transplanting pig organs into non-human primates, a significant reduction of the survival time was observed when the organs were PCMV-infected. The possible mechanisms of reduction of survival time are based on the disruption of the coagulation system and by suppression and exhaustion of the immune system. Although PCMV is resistant against antivirals which are effective against other herpesviruses and although vaccines against PCMV do not exist, PCMV can easily eliminated by early weaning and isolation of the negative animals.

## Abbreviations

BaCMV: Baboon cytomegalovirus
CC: Consumptive coagulopathy
CRISPR/Cas: Clustered regularly interspaced short palindromic repeats/ CRISPR associated
GVHD: Graft versus host disease
HCMV: Human cytomegalovirus
HEV: Hepatitis E virus
HHV-5: Human herpesvirus 5
HHV-6A: Human herpesvirus 6A
HHV-6B: Human herpesvirus 6B
HHV-7: Human herpesvirus 7
ICAM-1: Intercellular adhesion molecule 1
MCMV: Murine cytomegalovirus
NIV: Nipah virus
PERV: Porcine endogenous retroviruses
PCV1: Porcine circovirus 1
PCV2: Porcine circovirus 2
PCV3: Porcine circovirus 3
PLHV-1, PLHV-2, PLHV-3: Porcine lymphotropic herpesviruses 1,2,3
PRRSV: Porcine reproductive and respiratory syndrome virus
TF: Tissue factor
